# Implementation of the Dementia Isolation Toolkit in long-term care improves awareness but does not reduce moral distress amongst healthcare providers

**DOI:** 10.1186/s12913-024-10912-5

**Published:** 2024-04-18

**Authors:** Anne Marie Levy, Alisa Grigorovich, Josephine McMurray, Hannah Quirt, Kaitlyn Ranft, Katia Engell, Steven Stewart, Arlene Astell, Kristina Kokorelias, Denise Schon, Kevin Rogrigues, Mario Tsokas, Alastair J. Flint, Andrea Iaboni

**Affiliations:** 1 KITE Research Institute, Toronto Rehabilitation Institute, University Health Network, Toronto, Ontario Canada; 2 Lazaridis School of Business & Economics, Wilfrid Laurier University, Brantford, Ontario Canada; 3 Recreation and Leisure Studies, Brock University, St. Catharines, Ontario Canada; 4 Department of Occupational Sciences & Occupational Therapy, University of Toronto, Toronto, Ontario Canada; 5 School of Psychology & Clinical Language Sciences, University of Reading, Berkshire, UK; 6 Department of Geriatrics, Sinai Health and University Health Network, Toronto, Ontario Canada; 7 Chair of Family Council, Lakeside Long Term Care Centre, Toronto, Ontario Canada; 8 Ontario Health Central, Toronto, Ontario Canada; 9 Department of Psychiatry, Temerty Faculty of Medicine, University of Toronto, Toronto, Ontario Canada; 10 Centre for Mental Health, University Health Network, Toronto, Ontario Canada

**Keywords:** Person-centred care, Moral distress, Long-term care, COVID-19, Dementia, Dementia isolation toolkit, Registered nurse, Nursing, Registered practical nurse, Personal support worker

## Abstract

**Background:**

Healthcare providers may experience moral distress when they are unable to take the ethically or morally appropriate action due to real or perceived constraints in delivering care, and this psychological stressor can negatively impact their mental health, leading to burnout and compassion fatigue. This study describes healthcare providers experiences of moral distress working in long-term care settings during the COVID-19 pandemic and measures self-reported levels of moral distress pre- and post-implementation of the Dementia Isolation Toolkit (DIT), a person-centred care intervention designed for use by healthcare providers to alleviate moral distress.

**Methods:**

Subjective levels of moral distress amongst providers (e.g., managerial, administrative, and front-line employees) working in three long-term care homes was measured pre- and post-implementation of the DIT using the Moral Distress in Dementia Care Survey and semi-structured interviews. Interviews explored participants’ experiences of moral distress in the workplace and the perceived impact of the intervention on moral distress.

**Results:**

A total of 23 providers between the three long-term care homes participated. Following implementation of the DIT, subjective levels of moral distress measured by the survey did not change. When interviewed, participants reported frequent experiences of moral distress from implementing public health directives, staff shortages, and professional burnout that remained unchanged following implementation. However, in the post-implementation interviews, participants who used the DIT reported improved self-awareness of moral distress and reductions in the experience of moral distress. Participants related this to feeling that the quality of resident care was improved by integrating principals of person-centered care and information gathered from the DIT.

**Conclusions:**

This study highlights the prevalence and exacerbation of moral distress amongst providers during the pandemic and the myriad of systemic factors that contribute to experiences of moral distress in long-term care settings. We report divergent findings with no quantitative improvement in moral distress post-intervention, but evidence from interviews that the DIT may ease some sources of moral distress and improve the perceived quality of care delivered. This study demonstrates that an intervention to support person-centred isolation care in this setting had limited impact on overall moral distress during the COVID-19 pandemic.

**Supplementary Information:**

The online version contains supplementary material available at 10.1186/s12913-024-10912-5.

## Background

Guided by codes of conduct or professional standards, providers are required to exercise judgement and decision-making in the provision of care. However, due to real or perceived constraints over which they have little control, providers may be prevented from doing what they believe is ethically or morally right [1]. Such events are considered moral stressors [2] that may precipitate moral distress; the emotional response experienced when prevented from doing (or not doing) what one believes is morally right [3, 4]. The accumulation of multiple instances of moral distress can give rise to a sense that one’s core values and integrity are threatened, which puts providers at risk for moral injury [2]. Moral injury can affect providers’ personal and professional lives as they cope with internalized feelings of guilt, shame, negative self-appraisal, learned helplessness, and diminished self-efficacy [5–8], and prolonged moral distress can result in long-lasting mental health issues [9].

The frequency and severity of moral stressors in health care settings rose sharply during the COVID-19 pandemic due to resource scarcity and operational changes, such as visitation restrictions that led to people dying alone [10]. In long-term care homes (LTCHs), these challenges were compounded by the vulnerability of resident populations, who were at greater risk of severe infection resulting in higher rates of mortality and longer periods of restrictive infection prevention and control (IPAC) measures, compared to those in other health care or community settings [9, 11]. Prior to the pandemic, healthcare providers working in LTCHs experienced moral distress at least daily or weekly [12]. The onset of the pandemic not only exacerbated pre-existing stressors [9, 13], but also created novel risk factors for moral distress (e.g., frequent and extended periods of resident isolation, restrictions on family visitors). Presently, there are limited evidence-based interventions and strategies available to reduce levels of moral distress and alleviate its impact on the wellbeing of health care providers (referred to herein as providers). The purpose of this study was to characterize the experience of moral distress amongst providers working in LTCHs in the context of the COVID-19 pandemic and evaluate the impact of implementing an intervention, the Dementia Isolation Toolkit (DIT [14]; on self-reported levels of moral distress.

LTCHs were disproportionately impacted by the COVID-19 Pandemic. During waves one and two of the pandemic (March 2020 – August 2020 and September 2020 – February 2021, respectively), over 80,000 staff and residents of LTCHs in Canada were infected with COVID-19, representing 10% of all cases [15]. Over 21,000 Canadians died of COVID-19 during this period, and of those over 14,000 were residents of LTCHs, accounting for 69% of all COVID-19 mortality [15]. The urgency to protect vulnerable residents and staff who were disproportionately affected by COVID-19, led to rapid changes in legislation governing LTCH operations and prioritization of IPAC [16]. These changes primarily involved utilizing quarantine and isolation, screening programs, and social distancing as key mitigation strategies to slow the spread of the virus.

Over 87% of adults residing in LTC in Canada are living with dementia or some form of cognitive impairment [17] which makes it challenging for them to understand, remember, and comply with IPAC mitigation strategies. To more effectively ensure isolation guidelines are adhered to, measures such as restraint (e.g., using pharmacological management, physical restraint, or seclusion) have been employed; however, these strategies place LTCH residents at risk of serious harm, including death, falls, and other injuries [18]. Moreover, pandemic-related public health mandates can conflict with person-centred care principles that may guide care practices in LTC settings (e.g., shared decision-making, self-determination, personalization of care, support of social and emotional needs) [19]. This conflict can create ethical tensions between the obligation to act according to public health guidelines that aim to protect the safety of the collective while also supporting the autonomy of residents and staff. These tensions have strained providers’ sense of personal and professional integrity, and those working during the pandemic reported increased moral distress stemming from witnessing dehumanizing care practices (e.g., family separation by restrictive public mandates, residents dying alone) [20] and diminished quality of care [5, 6, 21].

There are few evidence-informed strategies to mitigate moral distress in healthcare providers. In general, the aim is to support the development of moral resilience which allows individuals to sustain or restore a sense of integrity when faced with complex ethical decisions [22]. Higher levels of moral resilience have been found to attenuate the negative effects of repeated exposure to moral stressors [8]. Developing competencies that build moral resilience through education and training programs also reduces healthcare providers’ levels of moral distress [1]. The Dementia Isolation Toolkit (DIT) [14] was developed by members of the research team in partnership with LTCH stakeholders to build these care competencies. The DIT consists of 1) an ethical guidance framework; 2) a person-centered isolation care plan worksheet; 3) an isolation decision-making worksheet; and 4) customizable isolation communication signage. It was developed to fill a need for plain language ethical guidance for providers working in LTC settings during the COVID-19 pandemic, to improve their ability to address: 1) the practical challenges of isolating or quarantining people with dementia in a compassionate, safe, and effective manner; and 2) the need for ethical guidance to support decision-making regarding infection control strategies, to alleviate moral distress and promote moral resilience amongst healthcare providers [14, 23]. The DIT aims to encourage person-centred care-planning during periods of outbreak, improve skills for delivering person-centred care, enhance communication within care teams, and increase the person-centredness of care for residents [23]. In a previous study conducted during the second wave of the Covid-19 pandemic (December 2020 to March 2021), an anonymous survey conducted amongst providers working in LTC, reported that job satisfaction was unaffected by moral distress for those using the DIT compared to those who had not used the DIT [23]. Further, roughly two-thirds of respondents found the DIT helpful for communicating decisions around care and developing isolation care plans, and about half reported that the DIT was helpful in reducing distress while providing care during the pandemic [23]. Respondents found the person-centered isolation care planning and isolation decision-making worksheet components of the DIT most helpful [23].

## Methods

### Study aim

This study aimed to characterize the experience of moral distress amongst providers working in three LTCHs during the COVID-19 pandemic, measure their self-reported levels of moral distress before and after the implementation of the DIT, and identify factors that may have influenced the impact of the DIT on moral distress.

### Design and setting

We employed a convergent parallel mixed methods study design, conducting pre- and post-implementation surveys and interviews with providers in three LTCHs located in urban or rural settings across southwestern Ontario, Canada (Table 1). The study took place between March and November 2021. The implementation of the DIT was led by a separate implementation team at each LTCH site comprised of up to four LTCH providers, one- or two-family care partners nominated by the LTCH to participate, and members of the research team. For the duration of the research study, the teams met bi-weekly, to co-design two training videos, develop the local implementation plan, and to provide real-time feedback and insights related to the implementation of the DIT (e.g., a monthly questionnaire, and transcripts of the bi-weekly team meetings).

**Table 1:** LTCH demographics

	Type	Population Size & Location	Beds	% Residents with Dementia as of 2020-21^a^	Duration of Implementation Study: March 2021 and November 2021			
					Total COVID-19 Cases: Staff ^b^	Total COVID-19 Cases: Residents ^b^	Total COVID-19 Related Deaths ^b^	% Staff Turnover ^b^
LTCH01	**Not for Profit**	**Large** **Population** **(Urban)**	**350**	**56.3%**	Unknown	Unknown	Unknown	Unknown
LTCH02	**Municipal**	**Medium Population** **(Urban)**	**126**	**66.0%**	10	1	0	18.8%
LTCH03	**Municipal**	**Small Population (Rural)**	**125**	**70.1%**	4	0	0	28.1%

Table one describes the characteristics of the long-term care homes (LTCH) that participated in the research study. Given limited resources and time, LTCH01 was unable to provide data related to staff turnover or COVID-19 related cases and deaths

^a^Data collected from CIHI Portal, Your Health System. Ottawa, ON: Canadian Institute for Health Information; 2022

^b^Data collected from the participating long-term care homes

The study design, using both quantitative and qualitative data and a temporal approach, was suitable for investigating the complex phenomenon of moral distress in a healthcare setting, and assessment of changes over time. Developmental evaluation (DE) has been recognized as a useful approach in implementation research [24], and during crises [25], and involves continuous feedback to the research team when complex and emergent conditions require community collaboration to understand and adapt studies implemented during high uncertainty. Foundational concepts of DE include relationship building, continuous learning and assessing and responding to context [25], that are manifested through processes such as flexible interventions that can adapt to emerging insights, and an emphasis on learning over time. Real-time feedback loops between the research and implementation teams were used to adjust the implementation plan to best support the dynamic and changing needs of the LTCHs. For instance, in response to the research team identifying six common first languages of front-line workers [26], two DIT training videos (~ 10-minutes, each) were subtitled in Chinese, Hindi, Portuguese, Spanish, Tagalog, Tibetan, and French [27] and disseminated via in-house employee learning platforms (Surge Learning) and on YouTube via the DIT website. The results of the developmental evaluation are beyond the scope of the research questions addressed in this paper.

### Study participants

Participants included any care provider working at one of the three LTCH sites. With the consent of their employers, participants completed surveys and interviews during or after work hours and subsequently received a $40 gift card. To preserve anonymity, participants did not have to request permission from a supervisor or manager to participate. To be included in the study, participants had to be fluent in English, employed at one of the three LTCH sites, and have physically worked there during the COVID-19 pandemic (i.e., after March 1st, 2020). Participants were excluded if they were not fluent in English or were not employed or working at one of the three participating LTCHs after March 2020. The point of saturation in qualitative health research, whereby minimal insight is gained from interviewing additional participants, is often reached between 6 and 12 interviews in homogenous groups [28]. Therefore, the study aimed to recruit between 6 and 12 participants from each site, to sufficiently evaluate the impact of the DIT on moral distress amongst providers working in LTC settings during the COVID-19 pandemic.

### Recruitment

The principal investigator (A.I) invited 12 LTCHs to participate in the research study because they had previously expressed an interest in learning more about the DIT intervention. Of those invited, three LTCHs indicated that they had the resources (e.g., staff and time) to commit to the study requirements and volunteered to participate in the study. Restrictions on in-person visits at these LTCHs necessitated the use of remote strategies to recruit participants into the research study. Recruitment was not randomized, and used convenience, purposive, and snowball sampling. Diverse recruitment methods were used, including remote introductory meetings hosted by the research team, email communications, and recruitment posters physically posted in the LTCHs. Prospective participants were invited via email, to participate in the research study and/−or to attend virtual introductory meetings with the research team to learn about the study and were required to self-identify their interest to participate with the research coordinator. Once a participant self-identified their interest to participate, they were required to read, sign, and submit their informed consent to participate in the research study online, via RedCap (electronic data capture tool hosted at the University Health Network).

### Data collection

Healthcare providers in each LTCH, regardless of participation in the research study, were invited through email (distributed by LTCH staff supporting the implementation of the research study) and informal communication routes (e.g., word of mouth, daily huddles, staff meetings) to complete training to use the DIT intervention. Training involved watching two DIT training videos (~ 10-minutes, each) that were available via in-house employee learning platforms (Surge Learning) or on YouTube via the DIT website. This training was voluntary. Electronic copies of the DIT were distributed to providers and physical copies of the DIT were available at the LTCH in spaces frequented by LTCH providers (e.g., nurses’ stations, lunchrooms). At the time of data collection, restrictions on in-person visits at LTCHs also necessitated remote data collection. Data were collected at two time points: at baseline prior to DIT implementation and again approximately 4–5 months following implementation of the DIT. Survey data were collected and managed using RedCap. Following submission of their informed consent form, participants were sent a link via email, to complete a demographic survey to collect information about the participants’ age, gender, highest education level attained, number of years in current profession/position, and characteristics of their role (e.g., managerial/administrative, frontline care), plus the Moral Distress in Dementia Care Survey (MDDCS [29];). This was followed by semi-structured, in-depth interviews conducted online with a member of the research team (A.L., K.R., or K.E.; See Supplementary Appendix A, Additional file 1 for samples of the interview questions). Prior to the start of each interview, participants were reminded of their right to skip any question they did not want to answer, that they could end the interview at any point, and that they could request their data be withdrawn from the study to ensure that participants retained control over the information that they chose to share.

### Instruments

#### The moral distress in dementia care survey

**Fig. 1:**
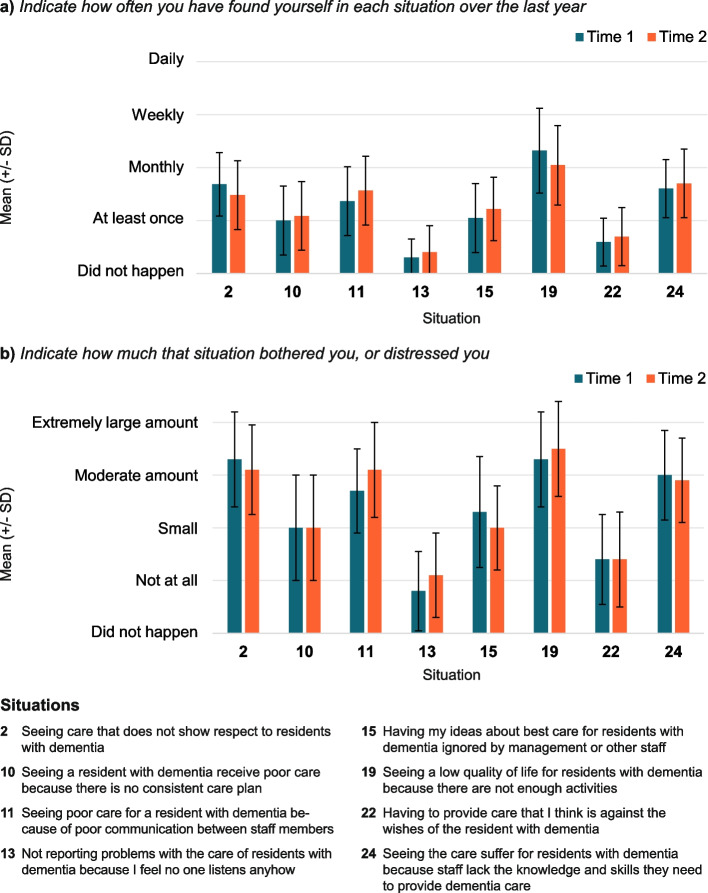
Participants’ (*N* = 23) mean (± standard deviation) responses to selected questions on the Moral Distress in Dementia Care Survey representing situations that the Dementia Isolation Toolkit (DIT) intervention was designed to target (numbers on the x-axis correspond to the situations described in legend). Situations were rated pre- and post-implementation

The MDDCS was the primary outcome measure in this study. The MDDCS is a validated, 56-item survey (taking approximately 15–20-minutes to complete) designed to measure moral distress in providers who care for persons living with dementia [12, 29]. Survey items are scored using Likert scales and are categorized into four subscales, including: 1. the frequency (1 = Has not happened in the last year, 2 = At least once, 3 = Once a month or more, 4 = Once a week or more, 5 = Daily) and 2. the severity (1 = Has not happened in the last year, 2 = None at all, 3 = A small amount, 4 = A moderate amount, 5 = A large amount, 6 = An extremely large amount) of 28 different situations that may trigger moral distress amongst providers, 3. the subjective effects of moral distress experienced by providers (1 = None, 2 = At least once, 3 = Once a month or more, 4 = Once a week or more, 5 = Daily)(19-items), and 4. the extent to which strategies may alleviate the impact of moral distress on providers (1 = None at all, 2 = A small amount, 3 = A moderate amount, 4 = A large amount, 5 = An extremely large amount) (8-items) [29]. Providers also used a 5-point Likert scale (1 = Not at all, 2 = A small amount, 3 = A moderate amount, 4 = A large amount, 5 = An extremely large amount) to indicate how much moral distress reduced their job satisfaction and made them want to quit their job [12, 29, 30]. For each Likert scale, a higher score (ranging from 1 to 6) indicated greater experience with or impact of moral distress on providers, and items within each subscale were analyzed separately (no aggregate score by subscale was utilized) [12]. Finally, providers were also asked to indicate whether they intended to quit their job within the next year (Yes or No) and rate the level of moral distress they experienced providing care to residents living with dementia over the past year, on a scale of zero (no moral distress) to 10 (level of moral distress that is too much to handle) [12, 29, 30]. The MDDCS broadly evaluates sources of moral distress that may be encountered in LTC settings. However, the DIT was designed to target situations that might cause moral distress specifically related to poor communication, inadequate care planning, delivering care that may not be in the best interest of residents, and decision-making related to care in the context of the COVID-19 pandemic. Therefore, for the analysis of the MDDCS subscales on frequency and the severity of situations that trigger moral distress, the 28-items were separated into two groups (determined by the research team) based on whether they were situations targeted by the DIT intervention (8 items; Fig. 1) or were morally distressing situations not directly targeted by the DIT (20 items; See Supplementary Table 1, Additional file 2).

#### Semi-structured interviews

Participants completed two 45–60-minute one-on-one interviews (pre- and post-implementation of the DIT) with members of the research team (A.L, PhD in Psychology specializing in Neuroscience and Applied Cognitive Science; K.E, MA in Recreation and Leisure; K.R, Master of Social Work) conducted online using Microsoft Teams. The interview guides included 8–10 open-ended questions that addressed participants’ experiences of moral distress working in LTC settings, the impact of moral distress on job satisfaction, participants’ use of the DIT, and how the DIT had an impact, if at all, on their levels of moral distress (See Supplementary Appendix A, Additional file 1). These interview data were triangulated with questionnaire data to supplement the analysis.

### Ethical considerations

Approval to conduct this research was obtained from both the University Health Network (#20–6262) and Wilfrid Laurier University (#6844) Research Ethics Boards. All methods carried out in the study were performed in accordance with relevant guidelines and regulations outlined in the Tri-Council Policy Statement: Ethical Conduct for Research Involving Humans.

### Data analysis

Quantitative survey data were analyzed using Statistical Package of the Social Sciences version 22 (SPSS v.22). These data were analyzed descriptively; frequencies or means with associated 95% confidence intervals were calculated, or medians and interquartile ranges if non-parametric statistics were required. Using Bayesian proportional odds models, we estimated an odds ratio, and 95% credible interval bounds of the posterior probability distribution for the post-implementation MDDCS responses compared to pre-implementation responses. Stata 16 was used to estimate the Bayesian statistics starting from uninformative priors.

Qualitative interview data were transcribed verbatim, anonymized, and transcripts were entered into NVivo 12 (QSR International). Interview data were analyzed using thematic analysis techniques [31], whereby members of the research team (A.G, A.I, A.L, J.M, K.E, K.R) read and re-read all qualitative data, developed a coding manual, and attached descriptive codes of analysis to segments of the texts. These descriptive codes were then grouped into broad, topic-oriented themes and over the course of three rounds of coding and discussions, the team negotiated differences in interpretation to finalize three themes.

## Results

Given the exploratory nature of this study and limited sample size (*N* = 23) due to recruitment challenges presented by the pandemic, findings from the quantitative analysis are presented as descriptive statistics. It is important to note, that the small sample size may restrict the generalizability of the results, and caution should be exercised in making broad inferences about the wider population. Formal statistical tests were conducted for descriptive purposes only, and the results should be interpreted with consideration of the study limitations.

### Participant demographics

Participants (*N* = 23, mean age 40.2 [SD = 11.1] and 87% female) had worked an average of 11.7 [SD = 10.2] years in the LTC sector (Table 2). At the time of the study, participants included providers in managerial/administrative (21.7%), registered nursing (8.7%), unregistered care (e.g., personal support workers; 8.7%), and allied healthcare roles (e.g., social workers, recreation, or behavioural support 60.9%), who had worked in these positions for an average of six [SD = 5.8] years. In the subsequent analysis of the qualitative interview data, participants are identified as either “administrative/managerial” or “frontline” (e.g., registered nursing staff, unregistered care staff, and allied healthcare) providers. There was no study attrition.

**Table 2:** Participant demographics

	Total	LTCH01	LTCH02	LTCH03
	**% (*N*** **= 23)**	39.1% (*n* = 9)	30.4% (*n* = 7)	30.4% (*n* = 7)
**Gender**				
Female	**87% (20)**	26.1% (6)	30.4% (7)	30.4% (7)
Male	**13% (3)**	13% (3)	0% (0)	0% (0)
**Age**				
20–29	**21.7% (5)**	4.3% (1)	8.7% (2)	8.7% (2)
30–39	**26.1% (6)**	13% (3)	4.3% (1)	8.7% (2)
40–49	**21.7% (5)**	13% (3)	4.3% (1)	4.3% (1)
50+	**21.7% (5)**	0% (0)	8.7% (3)	8.7% (2)
Unknown	**8.7% (2)**	8.7% (2)	0% (0)	0% (0)
**Education**				
College certificate or diploma	**43.4% (10)**	8.7% (2)	21.7% (5)	13% (3)
University certificate or diploma below bachelor level	**8.7% (2)**	4.3% (1)	4.3% (1)	0% (0)
Bachelor’s degree	**26.1% (6)**	8.7% (2)	4.3% (1)	13% (3)
Master’s degree	**17.4% (4)**	13% (3)	0% (0)	4.3% (1)
Unknown	**4.3% (1)**	4.3% (1)	0% (0)	0% (0)
**Job category**				
Managerial/Administrative	**30.4% (7)**	8.7% (2)	13% (3)	8.7% (2)
Frontline	**69.6% (16)**	30.4% (7)	17.4% (4)	21.7% (5)

Table 2 describes participant demographics for the overall research study in the Total column (*N* = 23) and broken down by long-term care home (LTCH01, 02 and 03). Note that in the bolded total column, the number of participants is reflected as a percentage of the total sample (*N* = 23).

### Moral distress in dementia care survey

Pre-implementation, participants often reported DIT-targeted situations of poor resident quality of care due to lack of activities, staff communication, and staff knowledge and skills (Fig. 1A). There were also several distressing situations not targeted by the DIT reported with moderate severity, including high staff turnover, a decline in care quality due to insufficient staff and families not fulfilling essential needs, and insufficient time and support to provide proper care (See Supplementary Table 1, Additional file 2).

For DIT-targeted stressor situations, there was no difference pre- and post-implementation in the frequency or severity of distress associated with the situations (Fig. 1 and Table 3). However, there was worsening distress related to several non-targeted situations after implementation of the DIT. In particular, participants experienced moral distress more often and were more bothered by being unable to ensure residents with dementia were in the right place to receive the right level of care (frequency OR 10.4; 95% CrI 2–85; distress OR 5.5; 95% CrI 1.2–25.8), and by the effect of staff turnover on the quality of dementia care (frequency OR 2.4; 95% CrI 0.6–9.9; distress OR 3.5; 95% CrI 1.1–12.5; See Supplementary Table 1, Additional file 2).

**Table 3:** The odds that healthcare providers experienced a change in the frequency and severity of moral distress in response to DIT-targeted situations post-implementation

			95% CrI^β^	
Description of situation		**OR**^**α**^	**Lower**	**Upper**
Seeing care that does not show respect to residents with dementia	Frequency	0.5	0.1	1.6
	Degree of distress	0.5	0.1	1.9
Seeing a resident with dementia receive poor care because there is no consistent care plan	Frequency	0.9	0.2	3.7
	Degree of distress	0.8	0.2	3.2
Seeing poor care for a resident with dementia because of poor communication between staff members	Frequency	2.7	0.6	14.4
	Degree of distress	2.1	0.5	7.7
Not reporting problems with the care of residents with dementia because I feel no one listens	Frequency	2.2	0.3	24.5
	Degree of distress	3.3	0.6	39.4
Having my ideas about best care for residents with dementia ignored by management or other staff	Frequency	1.8	0.4	7.3
	Degree of distress	0.5	0.1	2.0
Seeing a low quality of life for residents with dementia because there are not enough activities	Frequency	0.4	0.1	1.4
	Degree of distress	1.1	0.3	3.9
Having to provide care that I think is against the wishes of the resident with dementia	Frequency	1.4	0.3	7.3
	Degree of distress	0.7	0.2	3.0
Seeing the care suffer for residents with dementia because staff lack knowledge and skills, they need to provide dementia care	Frequency	1.2	0.3	4.6
	Degree of distress	0.8	0.2	2.9

^*α*^*Odds Ratio*

^*β*^*Credible Interval*

Table 3 displays the odds ratios (OR) and 95% credible interval (CrI) for participant responses to 8 questions from the Moral Distress in Dementia Care Survey that were selected by researchers, because they represent situations that the Dementia Isolation Toolkit intervention was designed to target in long-term care home (LTCH) settings. Participants responded to these 8 situations by indicating both, “How often have these situations occurred in the past year?” (Frequency) and “How much distress has this caused you?” (Degree of distress), on 5-point Likert scales.

**Fig. 2:**
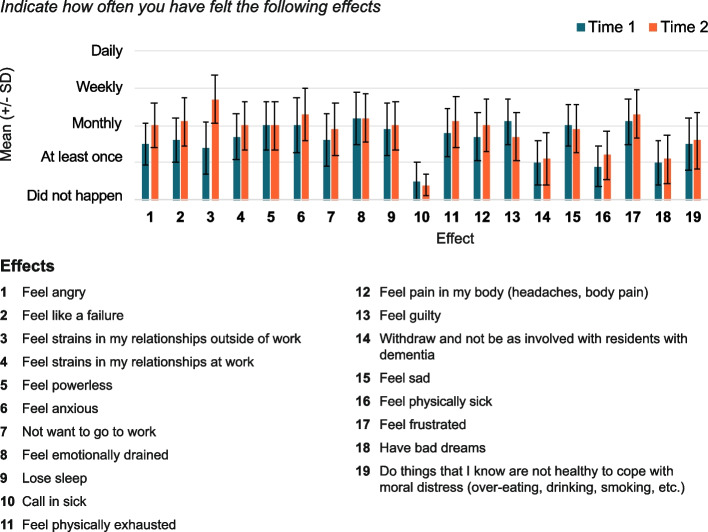
The impact of moral distress on participants (*N* = 23), with the mean (± standard deviation) of responses to 19 questions about effects of moral distress from the Moral Distress in Dementia Care Survey, rated pre- and post- DIT implementation

**Fig. 3:**
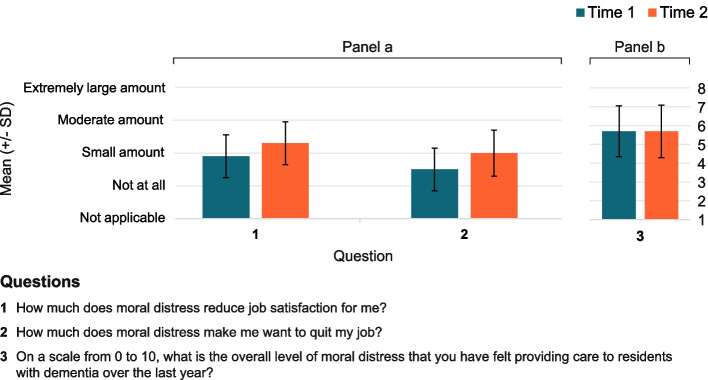
Participants (*N* = 23), mean (± standard deviation) responses to 3 questions from the Moral Distress in Dementia Care Survey pre and post DIT implementation.,a) Impact of moral distress on participants job satisfaction and desire to quit their job on a scale from 0 to 5; and b) rating of overall level of moral distress they experienced providing care to residents with dementia over the last year on a scale of 0–10 with0 being no moral distress and 10 being a level of moral distress that was too much to handle

In terms of the emotional effects of moral distress, participants initially reported experiencing these effects monthly. This included feelings of emotional exhaustion, guilt, and a sense of failure (Fig. 2). On average, participants initially rated their overall moral distress at 5.7 (SD = 2.7) out of 10, where 10 is too much to handle, and that moral distress negatively affected their job satisfaction “a small amount”, with a mean rating of 3.1 (SD = 1.3) out of 10 (Fig. 3). There was no statistical difference pre- and post-implementation in the overall level of moral distress (OR 1.0; 95% CrI 0.3–3.4), nor the effect of moral distress on job satisfaction (OR 3.0; 95% CrI 0.8–11.7). However, the effect of moral distress on providers’ desire to quit their job was greater post-implementation than pre-implementation (OR 4.9; 95% CrI 1.2–24.8) (Fig. 3). After implementation of the DIT, study participants reported feeling guilty less often (OR 0.1 95% CrI 0–0.7) but feeling anxious more often (OR 70.3 95% CrI 2.6–13,625.1). They also reported more physical exhaustion (OR 26.1; 95% CrI 2.1–1888.6), strains in relationships outside of work (OR 22.8; 95% CrI 2.0–889.2), and feelings of failure (OR 10.4; 95% CrI 2.0–85) and anger (OR 5.7, 95% CrI 1.5–27.1) compared to pre-implementation.

With respect to factors which influenced change in moral distress, there was no relationship found between age or years of experience. Compared to other roles, those in a managerial/administrative role were more likely to report increases in how often they felt strain at work (OR 2.4; 95% CrI 1.04–34.3), how bothered they were by staff turnover (OR 60.1; 95% CrI 1.2–9465), and how much moral distress made them want to quit their job (OR 5.0; 95% CrI 1.2–25.7). Overall, those with more job dissatisfaction pre-implementation reported a greater increase in feeling angry post-implementation of the DIT (OR 67; 95% CrI 7.6–195).

### Interviews

#### Moral distress in LTCH settings during the COVID-19 pandemic

To protect vulnerable populations in LTC settings during the Covid-19 pandemic, the provincial government of Ontario issued operational directives to LTCHs defining who was allowed to enter, the number of visitors per resident, and rules related to quarantine and isolation. However, it remained the responsibility of LTCH providers to enforce these directives. Participants reported that these operational changes made it challenging or impossible to deliver person-centred care to residents and identified that these and related challenges were common moral stressors.

“It was hard to have a limit on the number of visitors. Visitors not allowed to come was so distressful, the height of the moral distress…there is often more than one person that wants to see them and now they [providers] are trying to decide who gets to see them, mom, or dad” [P101, Frontline].

Participants expressed feelings of guilt and remorse when they were required to limit visitors and the amount of time those visitors could spend with residents who were at end of life, “That was the worst. It was awful. I definitely was teary and cried over some of those situations” [P306, Frontline]. Implementation of directives became increasingly stressful for providers, as they observed that the on-going isolation negatively impacted residents’ physical and psychosocial wellbeing. One participant stated, “I feel like it’s [isolation] almost one of the meanest things you can do to people” [P203, Frontline], and another suggested that the directives did not “make it difficult to provide physical care. It makes it difficult to provide the emotional care” [P303, Managerial/Administrative]. Social distancing between staff and residents was also mandated, which presented staff with daily opportunities to experience moral stressors when they were required to withhold physical comforts from residents.

“It was really hard when a resident would be scared or having a bad day or crying…it was just so strict and we had to be so careful not to break those rules, even though our hearts were pulling us in that direction” [P105, Frontline].

Participants explained that they bore the brunt of frustrations and anger expressed by residents and care partners related to visiting rules or the availability of recreational programming “It was exhausting to come into work every day. It was also a struggle for me... I didn’t like lying to people [about directives], so I wouldn’t. Then people would be mad at me” [P205, Frontline]. Participants also expressed that these responsibilities had led to a perceived loss of control over their ability to deliver person-centred care that they believed residents deserved.

“I think safety of the residents did take precedence over personalized care and making sure that people had a say. It was just, "What can we do to keep people safe?" It was almost like a survival mode where basic needs trump your wishes and wants” [P205, Frontline].

Amid the pandemic, participants perceived few opportunities to positively influence residents’ quality of life as part of their day-to-day care and reported a diminished sense of self-efficacy and confidence in their ability to do their job, “A few staff said they felt useless because they can’t do the program that they used to. They don’t feel effective” [P301, Managerial/Administrative]. Moreover, workplace instability, largely driven by the frequent changes in public health directives also added to participants’ sense of a diminished ability to influence positive change, “Things kept changing, so there was no consistency. It’s like, how do you make it better when every day looks a little different?” [P101, Frontline]. In these morally stressful situations, participants reported that they protected their sense of integrity by breaking rules. These situations arose when staff felt compelled to bend rules to minimize perceived harm to residents and practice person-centred care, “To be honest, there were times when people didn’t follow the rules…imagine having two spouses that they couldn’t get together at the same facility and one is dying and the other one can’t come say goodbye” [P303, Managerial/Administrative]. Participants did, however, note that the decision to defy directives was not without consequence, self-reporting anxiety and fear of being caught and reprimanded.

“If I didn’t do anything, then I felt like I was neglecting the residents. If I did something it also put me in the "catch-22" situation where I was like, "Oh, I'm not following the directives…I felt like we had to be very secretive” [P109, Frontline].

Unprecedented and chronic understaffing was cited as a primary cause of moral distress and participants’ perceived inability to deliver quality care. Participants explained that this put pressure on them to meet standards of care and productivity that were unrealistic given staffing ratios, and this led to feelings of guilt and inadequacy. For example, “I feel like there’s just not enough of me or enough hours to try to get to everybody” [P305, Frontline] or “There’s never enough of me to provide the care” [P207, Frontline]. Under these constraints, participants reported they were forced to pick and choose who received care. They observed that while all residents received some level of care it was “maybe not the best care. I think everyone did get the care, but just maybe not the care they deserve” [P304, Frontline]. Participants also reported that working short staffed created moral stressors that negatively impacted care delivery. They often shouldered the blame themselves, “If the staff are stressed, it really rolls off. I feel like all the other residents, whether they have dementia or no dementia, they can feel it. It affects just your care that you’re delivering to the resident” [P109, Frontline].

Similarly, inadequate staffing adversely affected workplace culture and expectations around work. A combination of external and self-imposed pressures to work above and beyond providers’ agreed responsibilities to bridge the gap in staffing became the norm. Participants reported they were unable to have vacations approved, they rarely made it through a break without being called back to work and felt pressure to remain at work while ill. Participants were also required to assume unconventional responsibilities to ensure adequate staffing, “We have no staff half the times, so we’re all wearing many, many hats. We help each other out because we know what’s the best for the residents” [P305, Frontline]. These conditions of work also limited participants’ abilities to care for themselves, which they believed diminished the quality of care they delivered and contributed to their moral distress.

“I think the guilt that people would feel not coming into work or taking a mental health day and that sort of thing, you couldn't. You just couldn't do it because you knew that they were short-staffed and that they needed your help, or that people were in their rooms, and they were isolated. I think staff put a lot of pressure on themselves to just go a million miles an hour and not think about taking care of themselves” [P105, Frontline].

Participants reported that chronic overwork and moral distress resulted in an overwhelming sense of burnout, affecting both their personal and professional lives. They expressed that these challenges compromised their capacity to provide what they considered to be adequate care. For example, “I think just because we’re just so burnt out and tired, we don’t have the energy to laugh and joke with them [residents]” [P304, Frontline]**.** Participants explained their capacity to do anything more than meet the basic care needs of residents was limited by burnout, and self-reported feelings of guilt and shame, “I’m at a point of burnout right now. I truly am. I don’t have the zest, or the compassion, or the fun, or the bubbliness, or any of that now” [P202, Frontline]. Burnout was likewise associated with diminished job satisfaction, in some instances participants resigned from their positions, “I think there was a lot of fear and guilt, especially working in this setting. Then obviously I have resigned. It [moral distress at work] did lower job satisfaction” [P205, Frontline]. Others attributed the impact of burnout to their desire to leave the LTC sector.

“Feeling the stress and the anxiety and the exhaustion that I'm feeling now, it has crossed my mind to go work at Walmart, to go work at Tim Hortons because it is so much on us and I feel like I'm always, always hitting that brick wall” **[**P207, Frontline].

Participants noted a reduction in the frequency of moral stressors and extent of moral distress when the LTCHs had the capacity to hire personnel for newly created IPAC roles (e.g., screeners) or to support resident care. Participants also explained that they felt moral distress was similarly alleviated when working in LTCHs that had single occupancy rooms or the infrastructure to dedicate a subset of rooms to quarantine and isolation. The ability to isolate residents on their own when required, reduced the number of people requiring isolation, such as newly arriving residents, and those who were symptomatic, returning from an excursion, or tested positive for COVID-19.

#### Impacts of using the DIT

At each LTCH, limitations on the providers’ ability to use the DIT were identified (see section 3.3.3), and negatively influenced the DIT’s impact on day-to-day tasks of providers. When the DIT was used, providers from each LTCH indicated that it was a valuable resource for addressing issues related to moral distress and the quality of care delivered to residents. DIT education and training helped participants label their psychological experiences as moral distress, to identify and communicate with colleagues about their symptoms, and appreciate how conditions in the workplace contributed to moral distress.

“When someone hears that there's this thing [moral distress] that is more generally being felt by people in certain jobs, and it's got a name and that people are actually feeling that…Sometimes coming out with a label can validate peoples experiences because some can’t sleep because they are thinking about these decisions they have to make at work… this wouldn’t have been discussed without the DIT” [P301, Managerial/Administrative]

Two of the participating LTCHs digitized components of the DIT and created permanent tabs in the residents electronic care plan under “Personhood” and “Isolation”. Participants believed integration of the personhood and isolation tabs into the resident’s existing care plan was an example of culture change and commitment to person-centred care even amid the on-going pandemic, “Who they [residents] are as a person is the most important thing, and it’s the first thing in the care plan…For me, it’s like this huge change. That’s a huge cultural change. That to me is impressive” [P307, Frontline]. Participants reported that the information collected in the person-centred isolation care plan and decision-making worksheets improved their ability to deliver personalized care. This improvement extended beyond physical needs to include the social and emotional needs of residents during onboarding and periods of quarantine or isolation.

“The DIT care plan not only gives the general information of the resident, but very specific ones, so that it's almost like a cheat sheet for new staff… If you look at the DIT care plan, it's almost like, "Oh, if I rely on this, I think I would be able to at least communicate with the resident and see their needs, the high-risk area, and what I can do to help them” [P102, Managerial/Administrative].

In these, and other examples, utilizing components of the DIT had a self-reported positive effect on the participants’ proficiency in task completion and their ability to provide better quality of care to residents. Participants felt these outcomes eased their experiences of moral distress in the workplace.

“I knew how busy the staff were, so when I wasn't there on weekends…I knew the residents were just in their rooms, not being checked on as often. I'm a very guilty person… I didn't even want to leave for the weekend… for them [providers] to just have that information in detail at their fingertips related to isolation, I was like, Okay, I don't feel as stressed out about leaving her there by herself” [P105, Frontline].

The implementation of the DIT, coupled with related education and training, helped re-prioritize principles of person-centred care in their daily care routines despite the on-going operational changes in LTC brought on by the pandemic.

“It really helped us try to change our view to be less clinical and more resident-focused on what they needed to survive the isolation, I guess. We stopped looking at it more like, we need them to be in isolation, and more like, what do they need to be able to be in isolation?” [P303, Managerial/Administrative].

#### Barriers to utilizing the DIT

Across all sites, participants reported little engagement with the ethical guidance tool, and frontline staff suggested this component was less useful because of their perceived lack of control over daily tasks and decision-making.

“I am not sure if there is a lot of room for each individual to put their unique feelings into it…If the residents have been exposed and they are going into isolation, it is not our role to be making decisions about this” [P302, Frontline]

Moreover, participants suggested that personal beliefs and values may have deterred engagement with the ethical guidance tool.

“I would say most of them [providers] have a general knowledge of ethics, but it’s also a sensitive topic and that some people might not feel comfortable talking about it depending on their personal value and their own priorities.” [P102, Managerial/Administrative].

Finally, participants expressed concerns over their ability to find the time to engage with any components of the DIT that required reflection on ethical decision-making, given chronic understaffing and limited time to complete the minimum requirements of their job.

“You do have to sit down and start questioning and challenge yourself perhaps to question yourself whether you've been doing your job right or wrong, and that's a big deal…They [providers] just don't have the time to do that, to read through it and discuss it with their colleagues and reflect on. I don't think they even have a moment to reflect on what they did that day.” [P102, Managerial/Administrative].

Even when participants recognized the value of reflective practices for addressing moral distress, some felt that utilizing the DIT felt insurmountable in the context of their workload in LTC during the pandemic.

“It is such a great initiative. In theory, it would have been amazing to have. I think it [the DIT] just felt like work. Extra work. Because I'm like, "I don't know if anyone's going to use it, but I feel like I should do it” [P301, Frontline].

## Discussion

In this study, we describe the pervasiveness of moral distress in different LTCHs during the COVID-19 pandemic, across both frontline and managerial/administrative providers, and across different phases of the pandemic. Implementation of the DIT in March 2021 had no impact on the frequency or severity of distress as measured by the MDDCS related to DIT-targeted situations, and no impact on the overall severity of moral distress. Post-implementation of DIT, participants reported feeling less guilty, but also more anxious, angry, and like a failure. In interviews, participants reported daily moral stressors stemming from responsibilities for implementing public health directives that they perceived were not in the best interest of residents physical or psychosocial well-being and chronic staffing shortages that worsened during the study, all of which negatively impacted resident care. The staffing shortage also limited participants ability to care for themselves at work, which had a knock-on negative impact on the quality of care delivered. In the interviews following the DIT implementation, participants self-reported improved awareness of moral distress in the workplace. They also self-reported reductions in the experience of moral distress and participants related this to feeling that the quality of resident care they were providing was improved by integrating principles of person-centred care and information gathered from the DIT.

There are several likely factors contributing to the lack of effect of the DIT implementation on moral distress as measured by MDDCS in this study. The effect of the pandemic unfolded in LTCHs over several phases, with different restrictions and challenges, and different evolving situations that impacted on moral distress. The timing of the study was such that it took place as vaccines were being widely rolled out in LTCHs in Ontario, with a reduction in the number, size, and mortality rate of COVID-19 outbreaks over the summer of 2021 [32]. Without ongoing outbreaks, the opportunities for the LTCHs to put the DIT tools into use were limited and the urgency to apply these care principles was reduced. At the same time, a new crisis was emerging: LTCHs were faced with terminating unvaccinated staff, exacerbating the existing staffing crisis, and were under increasing pressures to admit patients from acute care hospitals [33]. These pressures are reflected in the increases in morally distressing situations and increased feelings of anxiousness, anger, or feelings of failure related to staff turnover, insufficient staff, and not being able to provide residents with the right level of care. Similarly, the stress and burden on the management/administrative staff, in terms of staff retention and recruitment, likely impacted the increased strain reported by this group over the study period. Overall, many of the contributing factors to moral distress, particularly those relating to individual provider’s and LTCHs’ control over care decision-making, went beyond the scope of what the DIT was intended to target, thus impacting on the effectiveness of the intervention.

Over the course of the study, LTCHs engaged primarily with the person-centred isolation care planning tool. Interviews demonstrated that participants connected the implementation of this tool to an improvement in person-centred care practices and this in turn, reduced subjective levels of moral distress reported in the interviews. This is in keeping with the existing literature in this area, that the provision of person-centred care is positively associated with healthcare provider-related outcomes, including job satisfaction [34, 35]. However, time constraints, lack of staffing, cost, educational gaps, poor teamwork, and lack of management support have all been shown to be barriers to person-centred care within LTC [36–39], consistent with barriers identified in this study. Interestingly, we found that there was limited to no engagement with the ethical guidance tool designed to improve awareness of ethical principles and decision-making to support identification of moral stressors and enable action to resolve moral distress. This educational tool was not effective at engaging participants, who identified a lack of power and time as a barrier to reflection and of raising and/−or addressing ethical questions or concerns. This is consistent with findings that low-control, low-reward, and emotionally demanding jobs such as front-line work in LTCHs are particularly at risk for moral distress [40].

Developing competencies that build moral resilience through education and training programs is an effective, evidence-based strategy to reduce providers’ levels of moral distress in pandemic and non-pandemic settings [1, 8, 41–43]. Interventions to support ethical competence and build moral resilience thus need to start at the level of culture and environment, creating time and space for ethical discussions and multidisciplinary participation in ethical decision-making [44]. Specific interventions such as ethics rounds and huddles are promising to help promote an ethical culture and help front-line staff exercise their moral agency [42, 43, 45]. The learnings of this study were used in the development of the “DIT Huddle” which was designed to overcome barriers to the use of the person-centred isolation care planning tool (e.g., too lengthy) and ethical guidance tool (e.g., lack of engagement). The “DIT Huddle” can be used to facilitate an interdisciplinary, brief stand-up meeting focused on an ethically challenging situation and it incorporates learnings from the ethical guidance tool, person-centered care planning and decision-making guidance tools included in the DIT. (https://dementiaisolationtoolkit.com/). More research is needed to understand whether the huddle is an effective approach for delivering the DIT. Additionally, in response to feedback from providers, a shorter version of the person-centred isolation care planning tool, named the isolation care plan summary, was developed and e-fillable digital versions of the DIT components were made available on the website to address the need for brevity and to provide more flexible options for data collection and communication between providers.

Our divergent findings (lack of evidence for improved moral distress in quantitative analysis but support for benefits in qualitative analysis) may be related to the small sample size and the MDDCS instrument used. The small number of participants was reflected in the large upper credible intervals for some analyses. A larger sample would reduce the uncertainty and variance of the posterior probability distribution. It is also possible, that the MDDCS may not have been sensitive and specific enough to detect changes in moral distress associated with the DIT. The pandemic has created novel sources of moral distress that have impacted the frequency, severity, and types of situations faced by participants, for example, the impact of implementing multiple and at times conflicting public health edicts. The MDDCS did not include items that captured these novel sources of moral distress created by the pandemic. There is a need for instruments that more accurately capture morally distressing situations related to outbreak situations (e.g., COVID-19 waves, influenza) and infection control and prevention more generally within LTC.

It is possible that using the DIT increased reporting of symptoms of moral distress by bridging an educational gap, heightening providers’ awareness of their moral distress, and/or enabling staff to label their experiences as moral distress and better appreciate the professional and personal consequences of this emotional response [46]. In support of this, managerial participants indicated in the post-implementation interviews that use of the DIT educated staff on the concept of moral distress. In the post-implementation interviews, participants did not report that improved awareness of the concept was associated with experiencing moral distress. Rather, they attributed the lack of change or increased moral distress and related outcomes (e.g., feelings of anger or failure) to specific factors unrelated to the DIT (e.g., chronic staffing shortages, having to follow care practices that they believed were not in the best interest of residents). In fact, in the post-implementation interviews the primary factor participants associated with reducing self-reported levels of moral distress was the perceived improved quality of care that they delivered to residents when they used the DIT intervention.

### Strengths and limitations

Data collection occurred during the third and fourth wave of the COVID-19 pandemic (March 2021–November 2021) and this enabled the evaluation of the DIT’s impact on providers levels of moral distress during a period of time when LTCH settings continued to have significant concerns related to IPAC brought on by the COVID-19 pandemic and the related LTCH-specific public health directives that led to frequent and disruptive operational changes. The mixed methods design allowed for the triangulation of survey and interview data both pre- and post-intervention which provided valuable insight into identifying the underlying factors that contributed to the lack of change in levels of moral distress over time and the interview data helped discern the positive effects of the intervention on levels of self-reported moral distress that were not measured by or detected by the survey. Using a developmental evaluation approach that was flexible and pragmatic to implement the DIT also allowed the implementation to be responsive to and address barriers to research in LTCHs posed by the COVID-19 pandemic. This approach enabled the participating LTCH’s and providers to support the study and use the DIT intervention despite limited resources (e.g. staff, time).

Taking a flexible and pragmatic approach, did however, result in variability between the sites in their approach to implementation that impacted the providers time and capacity to engage with training to use the DIT and/−or the extent to which the intervention was integrated within existing workflows at the LTCH. This approach also impacted the timing of data collection. For a subset of participants, pre-implementation surveys and interviews occurred after the DIT was made available for widespread use in the LTCH. It is possible, that a participant may have seen or reviewed the DIT prior to participating in the pre-implementation interview; however, none of the participants reported having used the DIT in their first interview. The staffing crisis severely limited the time providers had to dedicate to data collection and flexibility was required to enable their participation.

There were also barriers to implementation and recruitment for the research study due to public health directives restricting on-site visits in LTC settings. To address this, the research team partnered with providers at each LTCH who acted as on-site proxies to oversee the implementation of the DIT and recruitment strategy. This approach placed the burden of work to implement on providers in the LTCH and the implementation suffered (e.g. lack of engagement with the DIT) if no one at the LTCH had the time to champion the intervention. The lack of researcher presence on-site constrained the scope of recruitment, the ability to standardize training, and the implementation process.

The small sample size may have also limited the generalizability of the results of the quantitative analysis and caution should be exercised in making broad inferences about the wider population. While it was observed that the participants’ experience working through the pandemic was impacted by their professional roles, the sample was weighted towards participants in recreation and leisure roles and fewer in nursing and supportive care roles. The unprecedented staff shortages brought on by the pandemic, which worsened during the research study, negatively impacted study recruitment. A larger and more representative sample of all professionals in the LTCH settings would have strengthened this study.

## Conclusion

This mixed methods study exposes the pervasive nature of moral distress among long-term care providers during the COVID-19 pandemic. The findings underscore the multifaceted systemic and institutional factors that contribute to moral distress that can significantly impact the well-being of staff in these settings [47]. Despite the study’s small sample size, the combination of quantitative and qualitative data provides valuable preliminary evidence on the potential effectiveness of the DIT in mitigating moral distress among LTCH providers. These data also highlight the importance of including stakeholders’ voices in research. Stakeholders from each LTCH had the opportunity to inform how the DIT was used throughout the research study and this improved the quality, use and adoption of the intervention. Moreover, this approach gave providers the agency to adapt components of the DIT to better integrate principles of person-centered care into a variety of tasks (e.g., improving resident admissions processes). These findings suggest a complex interplay between the intervention and staff experiences, warranting further exploration with larger cohorts to validate and expand upon these initial observations.

Moving forward, it is imperative to recognize the pressing need for innovative strategies in delivering person-centered care interventions. This study highlights the adverse effects of depersonalized public health and infection control measures, emphasizing the urgency to identify approaches that minimize harm while promoting the well-being of both care providers and residents in long-term care settings. This research serves as a foundation for future investigations, encouraging comprehensive inquiries into effective interventions that prioritize the mental health and job satisfaction of those on the front lines of long-term care during emergencies.

### Supplementary Information

**Supplementary Material 1.**

**Supplementary Material 2.**

## Data Availability

Data generated by this research study may be requested from the corresponding author.

## Abbreviations

COVID-19: Coronavirus Disease 2019
DIT: Dementia Isolation Toolkit
DE: Developmental Evaluation
IPAC: Infection Prevention and Control
LTC: Long-term Care
LTCH: Long-term care home
MDDCS: Moral Distress in Dementia Care Survey
