# Supplementary material for: Implementation of the Dementia Isolation Toolkit in long-term care improves awareness but does not reduce moral distress amongst healthcare providers
Source: BMC Health Serv Res. 2024 Apr 18;24:481. doi: 10.1186/s12913-024-10912-5 (PMC11027277; doi:10.1186/s12913-024-10912-5)
Supplement: Supplementary file 1 — Supplementary Material 1. [file 12913_2024_10912_MOESM1_ESM.docx]

**Appendix A**

**Sample questions from the Pre-and Post-implementation semi-structured interview guide**

**Pre-implementation of the Dementia Isolation Toolkit**

- 1. In what way(s) were you involved in providing care for residents in isolation in your home?

1. Can you describe what isolation looked like for residents in your home?
2. What strategies did you use that were successful?
3. What strategies did you use that were unsuccessful?
4. How well do you feel you were able to achieve infection control for any residents who were not in isolation? Provide a safe environment for the isolated resident?
5. What challenges did you face in providing safe and compassionate isolation care for residents?
6. Can you think of any challenges related to your organization?
7. Can you think of any challenges related to external policies and directives?
8. How have these challenges changed throughout the course of the pandemic?
9. Can you think of any challenges related to the environment?
10. Can you think of any examples related to residents living with dementia?
11. Were there any strategies that helped you overcome these challenges?
12. In your opinion, were staff provided with the resources they needed to provide appropriate care for residents living with dementia in isolation? <Prompt: please explain your response>
13. Throughout the pandemic,
14. Have you noticed a change in the motivation or well-being of staff in the home? <Prompt: please explain your response>
15. Have you faced any rules or policies that go against what you feel is the right thing to do? <Prompt: please explain your response>

**Post-implementation of the DIT**

1. Have you provided direct care to residents in isolation since March 2021?
2. What challenges did you face in providing safe and compassionate isolation care for residents?
3. When you used the DIT,
   1. How did it, if at all, impact your ability to communicate about the resident’s isolation care plan with other staff? With their family caregiver?
   2. How were you most likely to communicate with other staff about the DIT?
   3. How has your ability to develop care plans for infection prevention/control for residents with dementia been impacted by using the DIT?
   4. How have your feelings of moral distress, if any, being impacted by using the DIT?
4. What barriers from within or external to your organization, did you face to provide safe, effective, compassionate isolation to your residents and their care partners? <Prompt: was this any different for residents living with dementia?
5. In your opinion, has your organization lived up to:
   1. Its goals as an organization? <Prompt: please explain your response>
   2. Its obligations to external stakeholders? <Prompt: please explain your response>
   3. Its obligations to staff? <Prompt: please explain your response>
   4. Its obligations to residents? <Prompt: please explain your response>
   5. Its obligations to care partners? <Prompt: please explain your response>
6. In your opinion, have the staff had:
   1. The resources to provide appropriate care for residents living with dementia in isolation? <Prompt: please explain your response>
   2. The motivation to provide appropriate care for residents living with dementia in isolation? <Prompt: please explain your response>
   3. To follow rules that they think compromise themselves, residents or care partners? <Prompt: please explain your response>
