# Supplementary material for: Implementation of the Dementia Isolation Toolkit in long-term care improves awareness but does not reduce moral distress amongst healthcare providers
Source: BMC Health Serv Res. 2024 Apr 18;24:481. doi: 10.1186/s12913-024-10912-5 (PMC11027277; doi:10.1186/s12913-024-10912-5)
Supplement: Supplementary file 2 — Supplementary Material 2. [file 12913_2024_10912_MOESM2_ESM.pdf]

Supplementary Table 1, Additional File 2. *Moral distress in the workplace*

a) *Indicate how often you have found yourself in each situation over the last year*

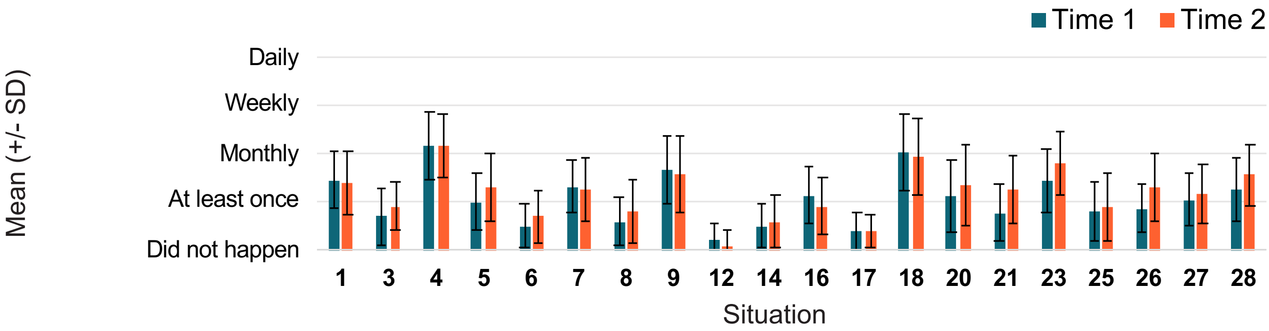

b) *Indicate how much that situation bothered you, or distressed you*

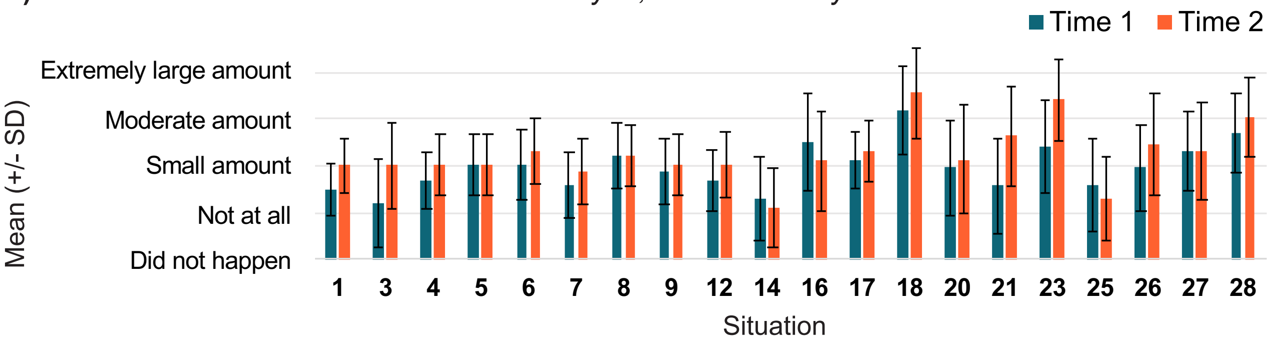

Situations

1 Having to follow a family's wishes for care even if it doesn't seem the best for the resident with dementia

3 Having to give care to the resident with dementia that I believe goes against the family's wishes

4 Telling the resident with dementia things that are not true so he/she won't get upset

5 Seeing residents with dementia get medication to control their behavior, even though I disagree with it

6 Carrying out doctor's orders that I don't think are the best for the resident with dementia

7 Seeing medications being withheld from a resident with dementia because that's what the family wants, but I believe the resident needs it

8 Avoiding giving care to a resident with dementia because I am afraid the resident might hurt me

9 Having to make a resident with dementia wait for care because another resident needs me just as much, at the same time

12 Not reporting what I believe is neglect or abuse of a resident with dementia because I'm afraid of causing trouble

14 Going along with care for a resident with dementia that I don't agree with because of pressure from my coworkers

16 Seeing residents with dementia living with pain because it is not treated appropriately

17 Having to give care to a resident with dementia that I believe goes against what is in their personal directive

18 Seeing the care suffer for residents with dementia because there are not enough staff to do the work

20 Having to rush the care of residents with dementia due to lack of time - even though I know it might upset them

21 Being unable to ensure that the resident with dementia is in the right facility to receive the right level of care

23 Seeing the care suffer for residents with dementia because of high staff turnover

25 Having to provide care to aggressive residents with dementia without the supports I need to feel safe

26 Having to work without the supports I need to prevent residents with dementia from hurting other residents

27 Seeing the care suffer for residents with dementia because physicians do not visit often enough

28 Seeing the care suffer for residents with dementia because families do not provide basic necessities such as clothing and other supplies

Legend

Supplementary Table 1, Additional file 2 depicts participants (N=23) mean (± standard deviation) response to questions on the Moral Distress in Dementia Care Survey that are designed to identify situations in the workplace that may precipitate moral distress. See Supplementary Table 1, Additional file 2 includes 20 of the 28 questions on this topic (numbers on the x-axis correspond to the situations described below the graphs). For each situation, participants indicated at Time 1 (pre-implementation) and again, 4-5 months later at Time 2 (post-implementation) of the Dementia Isolation Toolkit (DIT), "How often have you found yourself in each situation over the past year" (panel a) and "Indicate how much that situation bothered you, or distressed you?"(panel b), using a 5-point Likert scale depicted on the y-axis.
